# Prevalence and Prognostic Value of Cachexia Diagnosed by New Definition for Asian People in Older Patients With Heart Failure

**DOI:** 10.1002/jcsm.13610

**Published:** 2024-11-05

**Authors:** Takumi Noda, Emi Maekawa, Daichi Maeda, Shota Uchida, Masashi Yamashita, Nobuaki Hamazaki, Kohei Nozaki, Hiroshi Saito, Kazuya Saito, Yuki Ogasahara, Masaaki Konishi, Takeshi Kitai, Kentaro Iwata, Kentaro Jujo, Hiroshi Wada, Takatoshi Kasai, Hirofumi Nagamatsu, Tetsuya Ozawa, Katsuya Izawa, Shuhei Yamamoto, Naoki Aizawa, Ryusuke Yonezawa, Kazuhiro Oka, Junya Ako, Shin‐ichi Momomura, Nobuyuki Kagiyama, Yuya Matsue, Kentaro Kamiya

**Affiliations:** ^1^ Department of Rehabilitation Sciences Kitasato University Graduate School of Medical Sciences Sagamihara Japan; ^2^ Department of Cardiovascular Rehabilitation National Cerebral and Cardiovascular Center Suita Japan; ^3^ Department of Cardiovascular Medicine Kitasato University School of Medicine Sagamihara Japan; ^4^ Department of Cardiovascular Biology and Medicine Juntendo University Graduate School of Medicine Tokyo Japan; ^5^ Department of Rehabilitation, School of Allied Health Sciences Kitasato University Sagamihara Japan; ^6^ Division of Research ARCE Inc. Sagamihara Japan; ^7^ Department of Rehabilitation Kitasato University Hospital Sagamihara Japan; ^8^ Department of Rehabilitation Kameda Medical Center Kamogawa Japan; ^9^ Department of Rehabilitation The Sakakibara Heart Institute of Okayama Okayama Japan; ^10^ Department of Nursing The Sakakibara Heart Institute of Okayama Okayama Japan; ^11^ Division of Cardiology Yokohama City University Medical Center Yokohama Japan; ^12^ Department of Cardiovascular Medicine National Cerebral and Cardiovascular Center Suita Japan; ^13^ Department of Rehabilitation Kobe City Medical Center General Hospital Kobe Japan; ^14^ Department of Cardiology Nishiarai Heart Center Hospital Adachi Japan; ^15^ Department of Cardiovascular Medicine, Saitama Medical Center Jichi Medical University Shimotsuke Japan; ^16^ Cardiovascular Respiratory Sleep Medicine Juntendo University Graduate School of Medicine Tokyo Japan; ^17^ Department of Cardiology Tokai University School of Medicine Tokyo Japan; ^18^ Department of Rehabilitation Odawara Municipal Hospital Odawara Japan; ^19^ Department of Rehabilitation Matsui Heart Clinic Saitama Japan; ^20^ Department of Rehabilitation Shinshu University Hospital Matsumoto Japan; ^21^ Department of Cardiovascular Medicine, Nephrology and Neurology University of the Ryukyus Nishihara Japan; ^22^ Department of Rehabilitation Kitasato University Medical Center Kitamoto Japan; ^23^ Department of Rehabilitation Saitama Citizens Medical Center Saitama Japan; ^24^ Director, Saitama Citizens Medical Center Saitama Japan; ^25^ Department of Cardiovascular Biology and Medicine, Faculty of Medicine Juntendo University Tokyo Japan; ^26^ Department of Cardiology The Sakakibara Heart Institute of Okayama Okayama Japan; ^27^ Department of Digital Health and Telemedicine R&D Juntendo University Tokyo Japan

**Keywords:** Asia Working Group for Cachexia, cachexia, heart failure, malnutrition, prognosis, sarcopenia

## Abstract

**Background:**

The Asian Working Group for Cachexia (AWGC) proposed a new definition of cachexia; however, its impact on cachexia prevalence and overlaps with other conditions, such as sarcopenia and malnutrition, are unclear. We investigated these aspects and the prognostic value of cachexia based on the AWGC on mortality in older patients with heart failure (HF).

**Methods:**

This study was a secondary analysis of a prospective multicentre cohort, namely, the FRAGILE‐HF cohort study. Older (≥ 65 years) patients who had been hospitalized due to decompensated HF were enrolled. We assessed the presence/absence of cachexia based on the AWGC and Evans' criteria. Sarcopenia and malnutrition based on the Asian Working Group for Sarcopenia 2014 and the Global Leadership Initiative on Malnutrition criteria were also assessed to compare their prevalence and the overlaps between them. Patients were stratified in relation to the presence/absence of cachexia based on the AWGC criteria, and their mortality rates were compared.

**Results:**

Of the 861 enrolled patients (median [interquartile range] age, 80 years [73–85 years]; male, 58.9%), cachexia, as evaluated based on the AWGC and Evans' criteria, sarcopenia and malnutrition, was present in 74.1%, 36.2%, 20.6% and 55.2% of patients, respectively. AWGC‐defined cachexia was most common in the four conditions. All‐cause death events occurred in 153 (18.1%) patients in 2 years. AWGC‐defined cachexia (adjusted hazard ratio [aHRs], 1.442; 95% confidence interval [95% CI], 0.931–2.233; *p* = 0.101) was not associated with all‐cause mortality in older patients with HF after adjusting for other HF prognosis factors, such as the B‐type natriuretic peptide and the Meta‐Analysis Global Group in Chronic risk score, whereas cachexia evaluated based on Evans's criteria (aHRs, 1.547; 95% CI, 1.118–2.141; *p* = 0.009), sarcopenia (aHRs, 1.737; 95% CI, 1.214–2.485; *p* = 0.003), and malnutrition (aHRs, 1.581; 95% CI, 1.094–2.284; *p* = 0.015) was associated with all‐cause mortality.

**Conclusions:**

Three‐quarters of older patients with HF had cachexia as evaluated by the AWGC criteria, and this was not associated with a worse prognosis. As the new AWGC cachexia criteria will result in a significantly larger proportion of patients being diagnosed with cachexia, the implementation of the criteria in clinical practice requires further consideration.

**Trial Registration:** UMIN‐CTR unique identifier: UMIN000023929

**Abbreviations**
ASMI: appendicular skeletal muscle mass
AWGC: Asia Working Group for Cachexia
BNP: B‐type natriuretic peptide
MAGGIC risk score: Meta‐analysis Global Group in Chronic risk score
MUST: Malnutrition Universal Screening Tool

## 1 Introduction

Older patients with heart failure (HF) are inclined to suffer from cachexia, as they are affected by inflammation, anorexia, fatigue, malnutrition and muscle wasting (sarcopenia) [1]. A group of scientists and clinicians met in Washington, DC, for the cachexia consensus conference in 2006, and Evans et al. suggested that ‘cachexia, is a complex metabolic syndrome associated with underlying illness and characterized by loss of muscle with or without loss of fat mass’ [2]. Further, the European Society of Clinical Nutrition and Metabolism (ESPEN) has proposed that cachexia is not merely severe malnutrition but chronic disease‐related malnutrition with inflammation [3]. These definitions, which have been scrutinized for their validity and reliability to assess cachexia, have since been investigated in patients with HF [4, 5, 6].

The Asia Working Group for Cachexia (AWGC) recently disclosed a new definition of cachexia for Asian patients with chronic disease [7]. However, the prevalence and prognostic value of cachexia defined by the AWGC criteria in older patients with HF remains to be investigated. In this study, we aimed to compare the prevalence of cachexia defined by the AWGC criteria and its overlaps with cachexia evaluated by conventional criteria (Evans' criteria) [2], sarcopenia and malnutrition. We also investigated the association of AWGC‐defined cachexia with mortality based on data detailing the prevalence, overlaps and prognostic implications of physical and social frailties and cognitive dysfunction in hospitalized elderly patients with HF (FRAGILE‐HF) cohort study.

## 2 Materials and Methods

### 2.1 Study Design and Patient Population

This study was a secondary analysis of the data from the FRAGILE‐HF cohort study, a prospective multicentre observational study conducted at 15 hospitals in Japan [8]. We included older patients (≥ 65 years) with HF who were first admitted due to HF decompensation between September 2016 and March 2018. The exclusion criteria were as follows: (1) a history of heart transplantation or left ventricular assist device implantation; (2) chronic peritoneal dialysis or haemodialysis; (3) acute myocarditis; (4) B‐type natriuretic peptide (BNP) levels < 100 pg/mL or N‐terminal proBNP levels < 300 pg/mL on admission; and (5) data were not available. This study was conducted in accordance with the tenets of the Declaration of Helsinki and was approved by the ethics committees of each participating hospital. Written informed consent was waived by each patient under the Ethical Guidelines for Medical and Health Research Involving Human Subjects per the Japanese Ministry of Health, Labour and Welfare, as this study was observational and did not require invasive procedures or interventions. The information (e.g., inclusion and exclusion criteria and participating institutions) about this study were disclosed on the University Hospital Information Network (UMIN‐CTR, unique identifier: UMIN000023929) before patient enrolment commenced.

### 2.2 Assessments of Cachexia, Sarcopenia and Malnutrition

Cachexia was evaluated using the AWGC criteria [7] and Evans' criteria. According to the AWGC criteria [7], cachexia is defined by (1) a low body mass index (BMI) (< 21 kg/m^2^) or weight loss exceeding 2% for 3 to 6 months and (2) one or more of the following conditions: low handgrip strength (< 28 kg for men and < 18 kg for women), a high C‐reactive protein level (CRP; > 0.5 mg/dL) and anorexia. As data collection for this registry was conducted before the AWGC cachexia criteria were published, weight loss was investigated based on the 1‐year change used in Evans' cachexia criteria [2, 6]. To ensure consistency with the AWGC criteria regarding the period and degree of weight loss, we also conducted sensitivity analyses using definitions of weight loss exceeding 4% and 5% over 1 year. Weight loss was assessed using the current weight evaluated by medical staff and medical history interviews. Patients were asked to report their weight 1 year prior when their HF condition was stable (i.e., not undergoing decompensated HF or during acute treatment). The presence of anorexia was evaluated using the questionnaire as follows: limited food intake (< 70% of usual food intake) or poor appetite.

The assessments of cachexia using Evans' criteria [6], sarcopenia [9] and malnutrition [10] were based on previous reports from the FRAGILE‐HF cohort study. Briefly, cachexia was evaluated using Evans' criteria [6] as (1) a low BMI (< 18.5 kg/m^2^) or weight loss over 5% for 1 year and (2) three or more of the following conditions: low handgrip strength (< 28 kg for men and < 18 kg for women), a low mid‐upper arm muscle circumference (< 10th tile stratified by age and sex, which is based on the Japanese Anthropometric Reference Data 2001 [11]), anorexia, fatigue and an abnormal biochemical profile (increased CRP > 5.0 mg/L, decreased haemoglobin [Hb] < 12 g/dL and/or low serum albumin < 3.2 g/dL).

Sarcopenia was assessed using a low appendicular skeletal muscle mass (ASMI) and low handgrip strength (< 26 kg for men and < 18 kg for women) or walking speed (4 m walking speed ≤ 0.8 m/s for both sexes) based on the criteria of the Asia Working Group for Sarcopenia 2014 [9, 12]. ASMI was measured using bioelectrical impedance analysis (bc‐622, Tanita, Tokyo, Japan), and low ASMI was defined as ≤ 7.0 kg/m^2^ for men and ≤ 5.7 kg/m^2^ for women. Malnutrition was determined based on the Global Leadership Initiative on Malnutrition criteria [13]. We used the Malnutrition Universal Screening Tool (MUST) [14] to screen the risk of malnutrition at the outset. Malnutrition was diagnosed in patients who met a MUST score ≥ 1 and had a low BMI (< 18.5 kg/m^2^ for age < 70 years and < 20 kg/m^2^ for age ≥ 70 years), low ASMI (≤ 7.0 kg/m^2^ for men and ≤ 5.7 kg/m^2^ for women) or decreased body mass (> 10%) for a year. All patients met the etiologic criteria because we included patients who had been hospitalized for HF decompensation.

### 2.3 Covariates

Prior to discharge from the hospital, all the haemodynamically stable patients were evaluated via a baseline physical examination, blood sampling and echocardiography, and medication was prescribed. To consider basic patient information, the severity of the HF and HF prognostic factors, we made the three models for adjusting covariates. Model 1 consisted of age and sex; and Model 2 was Model 1 + the log‐transformed BNP and estimated glomerular filtration rate (eGFR); lastly, Model 3 included Model 2 + the Meta‐Analysis Global Group in Chronic (MAGGIC) HF risk score because it is an established HF prognostic factor.

### 2.4 Outcomes

We prospectively collected data on the patients' prognoses within 2 years of discharge. The primary outcome was all‐cause death. We also investigated the mode of death (i.e., cardiovascular and non‐cardiovascular death). We followed the patients at the respective hospitals at least every 3 months after discharge. In cases where we could not follow patients because they did not visit the hospital or clinic, we obtained the prognostic data from other medical facilities via telephone interviews to determine the clinical course of the patients.

### 2.5 Statistical Analysis

To investigate the overlaps between cachexia, sarcopenia, and malnutrition, an upset plot was drawn to evaluate their intersection size. We used the ‘UpSetR’ package, Version 1.4.0 (https://CRAN.R‐project.org/package=UpSetR), in R. The patients were divided into two groups based on the presence/absence of each condition, such as cachexia, sarcopenia and malnutrition, and their clinical characteristics were compared using the Mann–Whitney *U* test for continuous variables and the chi‐squared test for categorical variables. The continuous and categorical data were expressed as medians (interquartile range [IQR]) and frequency (%), respectively.

Cox regression analysis adjusted for covariates (Model 1: age and sex, Model 2: Model 1 + log‐transformed BNP and eGFR, and Model 3: Model 2 + the MAGGIC risk score) was used to calculate the hazard ratios (HRs) and 95% confidence intervals (CIs) to compare the mortality risk among the patients with cachexia determined using the AWGC and Evans' criteria, sarcopenia and malnutrition. We investigated the association between the overlap of each condition and mortality in the same analysis. Kaplan‐Meier survival curves and the log‐rank test were also used to investigate the significance of all‐cause mortality, comparing the prognosis of the number of overlaps of each condition. We used multiple imputations using the ‘mice’ package, Version 3.14.0, in R to complement the missing values and generated 20 datasets. All the analyses were conducted using R Studio statistical software (R Foundation for Statistical Computing, Vienna, Austria) Version 4.2.0. *p* < 0.05 were considered statistically significant.

## 3 Results

**FIGURE 1:**
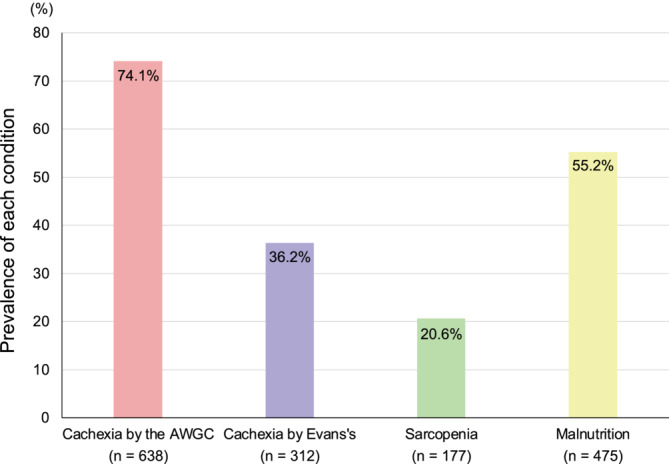
Prevalence of cachexia evaluated by the AWGC and Evans's criteria, sarcopenia and malnutrition in older patients with HF. AWGC, Asia Working Group for Cachexia.

**FIGURE 2:**
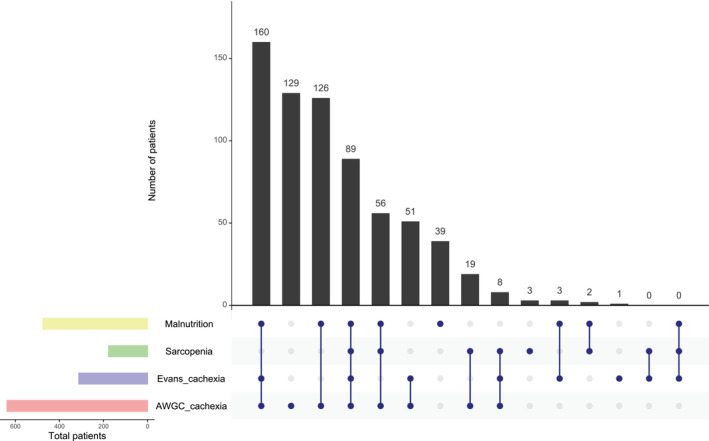
Upset plot detailing the overlap among cachexia based on AWGC and Evans's criteria, sarcopenia and malnutrition in 861 older patients with HF. AWGC, Asia Working Group for Cachexia.

The FRAGILE‐HF study prospectively enrolled 1332 hospitalized patients aged ≥ 65 years during the study period. In our study, we excluded 471 patients who were not evaluated for at least one condition due to missing diagnostic data. Finally, 861 patients were included in the analysis (Figure S1). The prevalence of cachexia evaluated by the AWGC and Evans' criteria, sarcopenia and malnutrition was 638 (74.1%), 312 (36.2%), 177 (20.6%) and 475 (55.2%), respectively (Figure 1). The prevalence of cachexia as defined by the AWGC criteria was higher than that of malnutrition; it was also twice the conventional incidence of cachexia determined by Evans' criteria. Figure 2 shows the overlaps between the four conditions. Eighty‐nine patients (10.3%) met the criteria for all four conditions, and 175 patients (20.3%) had no status. When comparing cachexia and the other conditions (i.e., sarcopenia and malnutrition), despite malnutrition being one of the most general conditions in cachexia, 32.4% of the cachexia patients assessed using the AWGC were not malnourished. Further, 28.2% of the AWGC cachexia cases existed independently without the coexistence of sarcopenia and malnutrition. In contrast, almost all patients diagnosed with cachexia according to Evan's criteria were also diagnosed with cachexia according to the AWGC criteria, and, in 223 patients who had no AWGC‐defined cachexia, the prevalence of patients with Evans's cachexia (4 patients [1.8%]), sarcopenia (5 patients [2.2%]) and malnutrition (44 patients [19.7%]) was low.

Table 1 and Table S1 show the characteristics of the patients for the four conditions. The patients in the AWGC cachexia group were older and had lower weight, BMI and mid‐upper arm and calf circumferences than those without cachexia as defined by the AWGC. Further, in the AWGC cachexia group, 127 all‐cause events occurred, and a higher event rate was present than in the AWGC non‐cachexia group; however, AWGC‐defined cachexia had no statistically significant association with cardiovascular death (*p* = 0.369). Cachexia by Evans' criteria, sarcopenia and malnutrition were associated with high all‐cause, cardiovascular and non‐cardiovascular deaths.

**TABLE 1:** Patient's characteristic.

	Missing data, *n* (%)	Overall	Cachexia by the AWGC			Cachexia by the Evans's			Sarcopenia			Malnutrition		
			No	Yes		No	Yes		No	Yes		No	Yes	
		*n* = 861	*n* = 223	*n* = 638	*p*	*n* = 549	*n* = 312	*p*	*n* = 684	*n* = 177	*p*	*n* = 386	*n* = 475	*p*
Age [years]	0	80 [73–85]	76 [71–82]	81 [74–86]	< 0.001	79 [72–85]	81 [75–86]	0.014	79 [73–85]	82 [77–87]	< 0.001	78 [72–84]	81 [74–86]	< 0.001
Male, *n* (%)	0	507 (58.9)	132 (59.2)	375 (58.8)	0.914	333 (60.7)	174 (55.8)	0.161	382 (55.8)	125 (70.6)	< 0.001	235 (60.9)	272 (57.3)	0.283
BMI [kg/m^2^]	0	20.8 [18.7–23.5]	23.2 [21.5–25.3]	20.0 [18.1–22.3]	< 0.001	21.7 [19.7–24.4]	19.2 [17.4–21.4]	< 0.001	21.5 [19.4–24.1]	18.4 [16.8–20.0]	< 0.001	23.1 [21.4–25.5]	19.0 [17.5–20.4]	< 0.001
Weight at baseline [kg]	0	52.6 [44.8–59.7]	58.2 [52.6–64.7]	50.2 [42.2–57.0]	< 0.001	55.0 [47.9–61.8]	47.4 [39.7–54.7]	< 0.001	54.5 [46.3–61.5]	46.5 [41.2–52.4]	< 0.001	58.1 [52.6–64.1]	46.6 [40.3–53.6]	< 0.001
Weight at 1 year prior [kg]	67 (7.8)	58.0 [50.0–65.0]	60.0 [53.8–67.0]	56.0 [48.0–65.0]	< 0.001	59.5 [52.0–65.0]	55.0 [46.9–64.0]	< 0.001	60.0 [51.0–66.8]	52.0 [46.0–58.0]	< 0.001	60.0 [55.0–66.9]	54.0 [45.0–64.0]	< 0.001
Weight loss rate over 1 year [%]	67 (7.8)	−6.6 [−11.6− −2.6]	−1.5 [−6.0–0.6]	−8.1 [−13.0− −4.3]	< 0.001	−4.2 [−8.2− −1.2]	−10.8 [−15.1− −6.9]	< 0.001	−6.1 [−10.8− −2.0]	−8.3 [−14.6− −4.3]	< 0.001	−4.1 [−6.7− −1.0]	−11.4 [−15.1− −5.9]	< 0.001
LVEF [%]	7 (0.8)	44.8 [32.0–59.6]	43.0 [30.3–58.0]	45.0 [32.0–60.0]	0.204	44.5 [32.0–59.0]	44.9 [31.0–60.0]	0.940	45.0 [32.1–59.0]	42.5 [30.0–62.0]	0.340	45.8 [34.0–60.0]	43.0 [30.0–58.9]	0.058
Heart failure phenotypes, *n* (%)	7 (0.8)				0.200			0.802			0.325			0.304
HFrEF (LVEF < 50%)		506 (59.3)	139 (62.9)	367 (58.0)		320 (58.9)	186 (59.8)		396 (58.4)	110 (62.5)		219 (57.3)	287 (60.8)	
HFpEF (LVEF ≥ 50%)		348 (40.7)	82 (37.1)	266 (42.0)		223 (41.1)	125 (40.2)		282 (41.6)	66 (37.5)		163 (42.7)	185 (39.2)	
Laboratory data at discharge														
CRP [mg/dL]	11 (1.3)	0.24 [0.10–0.72]	0.17 [0.07–0.33]	0.30 [0.11–0.85]	< 0.001	0.23 [0.09–0.69]	0.28 [0.11–0.81]	0.053	0.22 [0.99–0.69]	0.32 [0.14–0.89]	0.013	0.24 [0.10–0.77]	0.24 [0.10–0.69]	0.798
eGFR [mL/min/1.73m^2^]	1 (0.1)	54.7 [37.2–71.5]	54.3 [37.4–69.9]	54.7 [37.0–71.8]	0.590	56.0 [38.3–70.6]	51.9 [35.7–73.0]	0.465	54.1 [36.6–71.1]	55.6 [40.9–71.6]	0.435	53.9 [35.9–70.3]	55.2 [39.4–71.7]	0.479
BNP [pg/mL]	107 (12.4)	261.9 [128.4–479.9]	216.1 [101.6–399.1]	285.5 [145.1–502.6]	0.003	237.0 [115.6–435.8]	308.9 [166.4–560.4]	< 0.001	254.8 [121.2–464.4]	287.6 [161.8–505.1]	0.033	231.0 [109.1–431.8]	286.5 [152.1–503.9]	0.008
Albumin [g/dL]	20 (2.3)	3.5 [3.2–3.8]	3.7 [3.4–4.0]	3.4 [3.2–3.7]	< 0.001	3.6 [3.3–3.9]	3.3 [3.1–3.6]	< 0.001	3.5 [3.2–3.8]	3.4 [3.1–3.7]	0.005	3.6 [3.3–3.9]	3.4 [3.1–3.7]	< 0.001
Haemoglobin [g/dL]	1 (0.1)	11.8 [10.4–13.3]	12.0 [10.5–13.9]	11.7 [10.4–13.0]	0.015	12.1 [10.6–13.8]	11.4 [10.2–12.6]	< 0.001	11.8 [10.4–13.3]	11.9 [10.4–13.1]	0.806	12.0 [10.5–13.7]	11.7 [10.4–12.9]	0.016
Anorexia, n (%)	10 (1.2)	373 (43.8)	38 (17.0)	335 (53.3)	< 0.001	157 (29.0)	216 (69.9)	< 0.001	287 (42.4)	86 (49.4)	0.095	142 (36.8)	231 (49.7)	< 0.001
Calf circumference [cm]	11 (1.3)	30.7 [28.3–33.0]	32.6 [30.9–34.7]	30.0 [27.5–32.5]	< 0.001	31.4 [29.2–33.8]	29.2 [26.5–31.6]	< 0.001	31.4 [29.1–33.8]	28.2 [26.0–29.9]	< 0.001	32.5 [30.3–34.8]	29.2 [26.6–31.4]	< 0.001
Low mid upper arm muscle circumference, *n* (%)	23 (2.7)	126 (15.0)	18 (8.4)	108 (17.3)	0.002	40 (7.5)	86 (28.2)	< 0.001	93 (14.0)	33 (19.0)	0.103	29 (7.8)	97 (20.9)	< 0.001
Low appendicular skeletal muscle index, *n* (%)	54 (6.3)	222 (27.5)	18 (9.5)	204 (33.0)	< 0.001	116 (22.9)	106 (35.2)	< 0.001	45 (7.1)	177 (100)	< 0.001	42 (11.8)	180 (39.8)	<0.001
Frailty, *n* (%)	28 (3.3)	448 (53.8)	49 (22.5)	399 (64.9)	< 0.001	210 (39.8)	238 (78.0)	< 0.001	325 (49.0)	123 (72.4)	< 0.001	157 (41.6)	291 (63.8)	< 0.001
MAGGIC risk score [point]	11 (1.3)	26.0 [22.0–29.0]	24.0 [20.0–28.0]	26.0 [22.0–30.0]	< 0.001	25.0 [21.0–29.0]	27.0 [23.0–30.0]	< 0.001	25.0 [21.0–29.0]	27.0 [24.0–31.0]	< 0.001	24.0 [20.0–28.0]	27.0 [23.0–30.0]	< 0.001
All‐cause mortality, *n* (%)	14 (1.6)	153 (18.1)	26 (11.8)	127 (20.3)	0.005	79 (14.5)	74 (24.4)	< 0.001	101 (15.1)	52 (29.5)	< 0.001	46 (12.0)	107 (23.1)	< 0.001
Cardiovascular mortality, *n* (%)	14 (1.6)	82 (9.7)	18 (8.1)	64 (10.2)	0.369	41 (7.5)	41 (13.5)	0.005	55 (8.2)	27 (15.3)	0.004	23 (6.0)	59 (12.7)	< 0.001
Non‐cardiovascular mortality, *n* (%)	14 (1.6)	71 (8.4)	8 (3.6)	63 (10.1)	0.003	38 (7.0)	33 (10.9)	0.049	46 (6.9)	25 (14.2)	0.002	23 (6.0)	48 (10.4)	0.022

*Note:* Median [interquartile range]; *n* (%). Low mid upper arm muscle circumference, < 10th tile stratified by age and sex, which is based on the Japanese Anthropometric Reference Data 2001. Low appendicular skeletal muscle index, ≤ 7.0 kg/m^2^ for men and ≤ 5.7 kg/m^2^ for women.

Abbreviations: BMI, body mass index; BNP, B‐type natriuretic peptide; CRP, C‐reactive protein; eGFR, estimated glomerular filtration rate; HFpEF, heart failure with preserved ejection fraction; HFrEF, heart failure with reduced ejection fraction; LVEF, left ventricular ejection fraction; MAGGIC, Meta‐analysis Global Group in Chronic Heart Failure.

The Cox regression analyses adjusted for Model 1 and 2 for all‐cause death showed that cachexia based on the AWGC criteria (adjusted for Model 1, HR, 1.657; 95% CI, 1.076–2.551; *p* = 0.022, and Model 2, HR, 1.610; 95% CI, 1.043–2.485; *p* = 0.032) was associated with all‐cause mortality, but after adjusting for other HF prognostic factors (Model 3), it was not related to all‐cause deaths (HR, 1.442; 95% CI, 0.931–2.233; *p* = 0.101). On the other hand, cachexia evaluated by Evans' criteria (HR, 1.547; 95% CI, 1.118–2.141; *p* = 0.009), sarcopenia (HR, 1.737; 95% CI, 1.214–2.485; *p* = 0.003) and malnutrition (HR, 1.581; 95% CI, 1.094–2.284; *p* = 0.015) were associated with high all‐cause mortality rates (Table 2).

**TABLE 2:** Adjusted hazard ratios of cachexia, sarcopenia and malnutrition for all‐cause mortality.

		Incident rates (per 100 person‐years)	95% CI	Model 1			Model 2			Model 3		
				Adjusted HR	95% CI	*p*	Adjusted HR	95% CI	*p*	Adjusted HR	95% CI	*p*
Cachexia by AWGC criteria	No	6.5	4.3–9.6	1.000	[Reference]			[Reference]			[Reference]	
	Yes	12.3	10.3–14.7	1.657	1.076–2.551	0.022	1.610	1.043–2.485	0.032	1.442	0.931–2.233	0.101
Cachexia by Evans's criteria	No	8.4	6.6–10.4	1.000	[Reference]			[Reference]			[Reference]	
	Yes	15.3	12.0–19.2	1.794	1.302–2.471	< 0.001	1.699	1.230–2.347	0.001	1.547	1.118–2.141	0.009
Sarcopenia	No	8.7	7.1–10.6	1.000	[Reference]			[Reference]			[Reference]	
	Yes	19.6	14.6–25.7	1.951	1.378–2.761	< 0.001	1.913	1.341–2.730	< 0.001	1.737	1.214–2.485	0.003
Malnutrition	No	6.7	4.9–9.0	1.000	[Reference]			[Reference]			[Reference]	
	Yes	14.4	11.8–17.4	2.024	1.426–2.874	< 0.001	2.000	1.407–2.851	< 0.001	1.581	1.094–2.284	0.015

*Note:* Model 1: age and sex. Model 2: Model 1 + log‐transformed B‐type natriuretic peptide and estimate glomerular filtration rate. Model 3: Model 2 + the Meta‐Analysis Global Group in Chronic Heart Failure score.

Abbreviations: AWGC, Asian Working Group for Cachexia; CI, confidence interval; HR, hazard ratio.

For the sensitivity analysis, we modified the AWGC criteria to a low BMI of < 21 kg/m^2^, or 4% or 5% weight loss over a year. The prevalence of cachexia evaluated by the modified AWGC for the 4% [604 patients (70.2%)] and 5% versions [576 patients (66.9%)] was lower than that of the original AWGC cachexia; however, both the 4% (HR, 1.480; 95% CI, 0.977–2.242; *p* = 0.064) and 5% versions (HR, 1.459; 95% CI, 0.980–2.173; *p* = 0.063) were still not associated with all‐cause death after adjusting for covariates (Model 3).

Figure S2 and Table S2 illustrate the association of the overlaps of cachexia, sarcopenia and malnutrition with all‐cause death. The coexistence of several conditions, such as AWGC‐defined cachexia combined with sarcopenia and malnutrition, was associated with higher mortality in older patients with HF (*p* for trend < 0.001).

## 4 Discussion

This is the first study on the prevalence and overlaps of cachexia assessed by the AWGC criteria and other conditions, such as sarcopenia and malnutrition, in older patients with HF. The main findings were (1) 74.1% of patients had cachexia as defined by the AWGC criteria, which had the highest prevalence compared to the other conditions (i.e., cachexia by Evans criteria, sarcopenia and malnutrition), and (2) cachexia defined by the AWGC was not associated with mortality after adjusting for HF severity, whereas Evans' cachexia, sarcopenia and malnutrition had high mortality rates.

The prevalence of cachexia evaluated by the AWGC in this study was 74.1%; however, it is very high compared to the conventional prevalence of cachexia in patients with chronic diseases including HF (7%–46%) [15]. Previous studies in patients with chronic wasting disease (e.g., advanced cancer and end‐stage chronic kidney disease) have shown that 35%–83% of patients had AWGC‐defined cachexia [16, 17, 18, 19]. Although a previous cross‐sectional study on patients with HF who participated in outpatient cardiac rehabilitation has demonstrated that 30% of patients were AWGC‐defined cachexia, differences in the prevalence of AWGC‐defined cachexia between ours and the previous study may represent a difference in the population and AWGC‐defined cachexia at an earlier study may be underestimated because they did not have the CRP data [20]. The main conditions considered to coexist with cachexia are severe malnutrition and decreased muscle mass; however, in this study, the prevalence of cachexia as defined by the AWGC (74.1%) was higher than that of malnutrition (55.2%), and malnutrition and sarcopenia did not overlap with 32.4% and 73.0% of the patients in the AWGC cachexia group, respectively. In addition, the nutritional status (e.g., BMI, arm and calf circumference and the prevalence of low ASMI) of the patients with AWGC‐defined cachexia was better than that of the patients with cachexia based on Evans' criteria, malnutrition or sarcopenia, and AWGC‐defined cachexia was not associated with all‐cause mortality. These facts suggest that the new AWGC diagnostic criteria may lead to a diagnosis of cachexia in patients who do not conform to the previously consensus concept of cachexia [2, 3].

One possible reason that cachexia defined by the AWGC had a high prevalence and was not associated with mortality is that the weight criterion is too high for older patients with HF. The new definition of cachexia of the AWGC was made with consideration of the differences in body mass between Asian and Western populations because Asians have a lower BMI and a differential contribution of BMI to chronic diseases compared to Caucasians [7]. Yet, the criterion of a BMI < 21 kg/m^2^ in the AWGC definition is higher than those of Evans [2] and Fearon's criteria [21], which are often used for Western populations. The mean and median BMI of patients with cachexia in the review of the AWGC have been concluded to be around 21–22 kg/m^2^ (it is unclear whether the mean and median BMI were only for the cachexia group) and the low BMI (< 21 kg/m^2^) in the AWGC definition was decided based on those results [7]; however, it has simultaneously been demonstrated that the mean BMI of most intervention studies in older patients with cachexia is < 20 kg/m^2^ [7]. Konishi et al. reported that one‐third of Japanese patients with HF had a BMI < 20.3 kg/m^2^ [15]. Based on the results of a multicentre study designed to evaluate the recent trends in the clinical characteristics of Japanese patients with HF, the median BMI was reported to be 20.9 kg/m^2^ (IQR: 18.5–23.6 kg/m^2^) [22]. In a study of the East Asian populations, a BMI ≤ 20 kg/m^2^ was associated with a higher occurrence of cancer and cardiovascular events compared to those in the 22.6–25.0 kg/m^2^ [23]. Based on these considerations, it is possible that a BMI < 21 kg/m^2^ to diagnose cachexia in the AWGC criteria is excessively high for older adults with HF.

Our research team has previously reported that cachexia evaluated based on Evans' criteria was prevalent in 35.5% of older patients with HF [6]; however, we used a BMI of < 18.5 kg/m^2^ and weight loss of > 5% per year as the weight criteria given the differences between Asian and Western populations. Furthermore, we demonstrated that cachexia, as evaluated using Evan's criteria, was associated with increased mortality in older patients with HF [6]. However, mild weight loss of 2.0%–5.0% in patients with HF has been reported not to be related to adverse events [24, 25]. It may be more important to assess the nature of weight loss, such as skeletal muscle mass, and lean body mass, rather than evaluating the weight loss rate. The prevalence of cachexia in Japanese patients with HF varies widely due to differences in cachexia assessment; however, cachexia has been found to range from 9% to 33% [15], and it is possible that the AWGC criteria have highlighted other conditions, such as frailty or the very early stage of cachexia (pre‐cachexia), as being highly prevalent. Given that AWGC‐defined cachexia combined with sarcopenia and malnutrition were associated with increased mortality in this study, a more detailed assessment of muscle weakness and body compositions, such as skeletal muscle mass and lean body mass index, may be more important for cachexia assessment of patients with HF.

## 5 Limitations

We acknowledged several limitations of this study. First, this study included only older Japanese adults with HF. Cachexia evaluated based on the AWGC criteria may therefore be helpful for other populations, such as those with cancer, and further investigations are needed to determine whether AWGC‐defined cachexia can be applied to them. Second, although we have used the bioelectrical impedance analysis for assessing ASMI in sarcopenia assessments, we could not rule out the effects of the bodily fluids, such as oedema. Finally, many patients who could not be evaluated for cachexia, sarcopenia or malnutrition were excluded, such as patients with missing values for assessments or with cardiovascular implantable electronic devices, because they could not use the bioelectrical impedance device.

## 6 Conclusion

In our study, three‐quarters of the older patients with HF were found to have AWGC‐defined cachexia, which had a higher prevalence than Evans' cachexia, sarcopenia and malnutrition. In addition, cachexia defined by the AWGC was not associated with all‐cause death in older adults with HF. As the new AWGC cachexia criteria will result in a significantly larger proportion of patients being diagnosed with cachexia, the implementation of the criteria in clinical practice requires further consideration.

## Author Contributions

Takumi Noda, Emi Maekawa and Kentaro Kamiya contributed to conceptualization, data curation, formal analysis, investigation, methodology, project administration, resources, software and writing the original draft. Emi Maekawa and Kentaro Kamiya contributed to funding acquisition and supervision. All authors revised the manuscript, gave final approval and agreed to be accountable for all aspects of the work, ensuring integrity and accuracy.

## Ethics Statement

The study protocol was conducted in accordance with the tenets of the Declaration of Helsinki and was approved by the ethics committee of each participating hospital. Because this was an observational study that did not entail invasive procedures or interventions, written informed consent was not required under the Ethical Guidelines for Medical and Health Research Involving Human Subjects, per the Japanese Ministry of Health, Labour and Welfare. All participants were free to withdraw from the study at any time, and study information, including study objectives, inclusion and exclusion criteria and names of participating institutions, was posted on the University Hospital Information Network (UMIN‐CTR, unique identifier: UMIN000023929) prior to patient enrolment.

## Conflicts of Interest

Dr Kentaro Kamiya received funding from Eiken Chemical Co. Ltd. Dr Yuya Matsue is affiliated with a department endowed by Philips Respironics, ResMed, Teijin Home Healthcare and Fukuda Denshi, and received an honorarium from Novartis Japan, Bayer Japan and Otsuka Pharmaceutical Co. Dr Nobuyuki Kagiyama is affiliated with a department funded by Philips Healthcare, Asahi KASEI Corporation, Inter Reha Co. Ltd and Toho Holdings Co. Ltd. based on collaborative research agreements. Dr Takatoshi Kasai is affiliated with a department endowed by Philips Respironics, ResMed, Teijin Home Healthcare and Fukuda Denshi. The other authors declare no conflicts of interest.

## Supporting information


**Figure S1.** Patient flow chart.

**Figure S2.** Kaplan–Meier survival curves of overall survival rate in patients divided into five groups based on the number of overlaps of cachexia evaluated by AWGC and Evans’ criteria, sarcopenia, and malnutrition.AWGC, Asia Working Group for Cachexia.

**Table S1.** Patient characteristics.

**Table S2.** Cox regression analyses of the association of the coexistence of multiple diagnoses for the all‐cause death.

## Data Availability

Data described in the manuscript, code book and analytic code will be made available upon request pending application and approval.
