# Supplementary figures and images for: Prevalence and Prognostic Value of Cachexia Diagnosed by New Definition for Asian People in Older Patients With Heart Failure
Source: J Cachexia Sarcopenia Muscle. 2024 Nov 5;15(6):2660–8. doi: 10.1002/jcsm.13610 (PMC11634483; doi:10.1002/jcsm.13610)

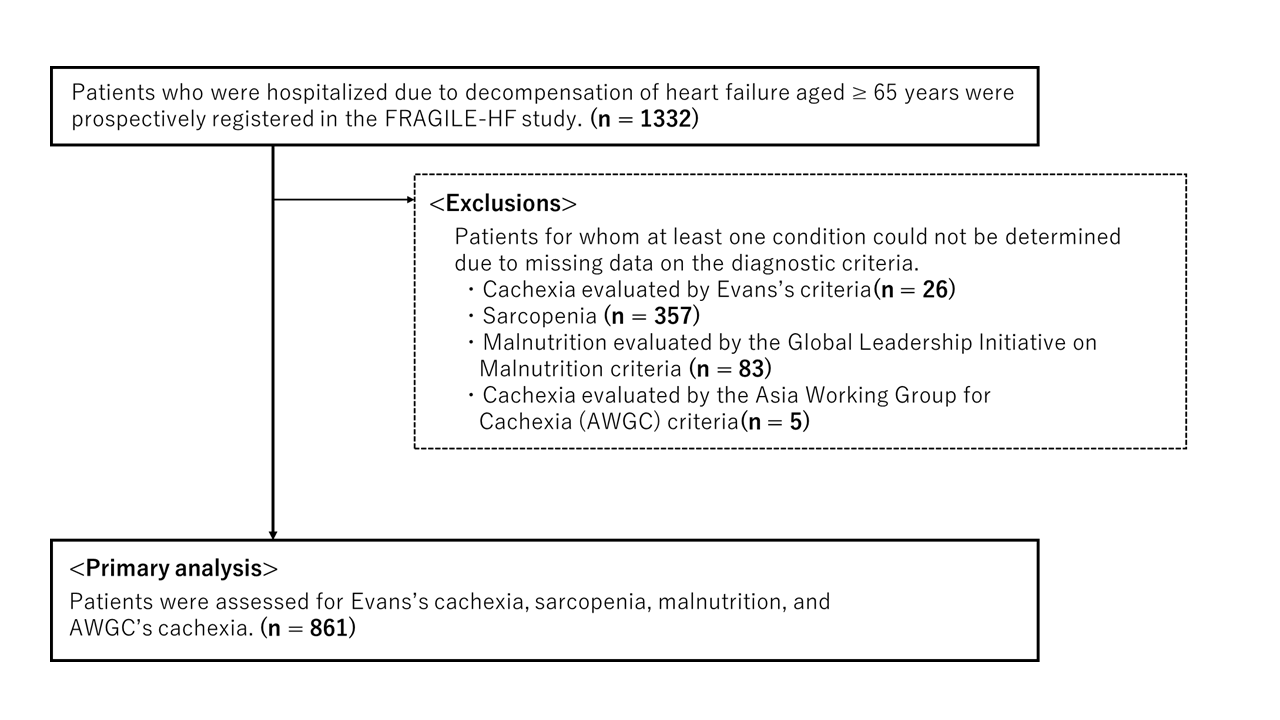

Supplement: Supplementary file 1 — Figure S1. Patient flow chart. [file JCSM-15-2660-s003.tif]

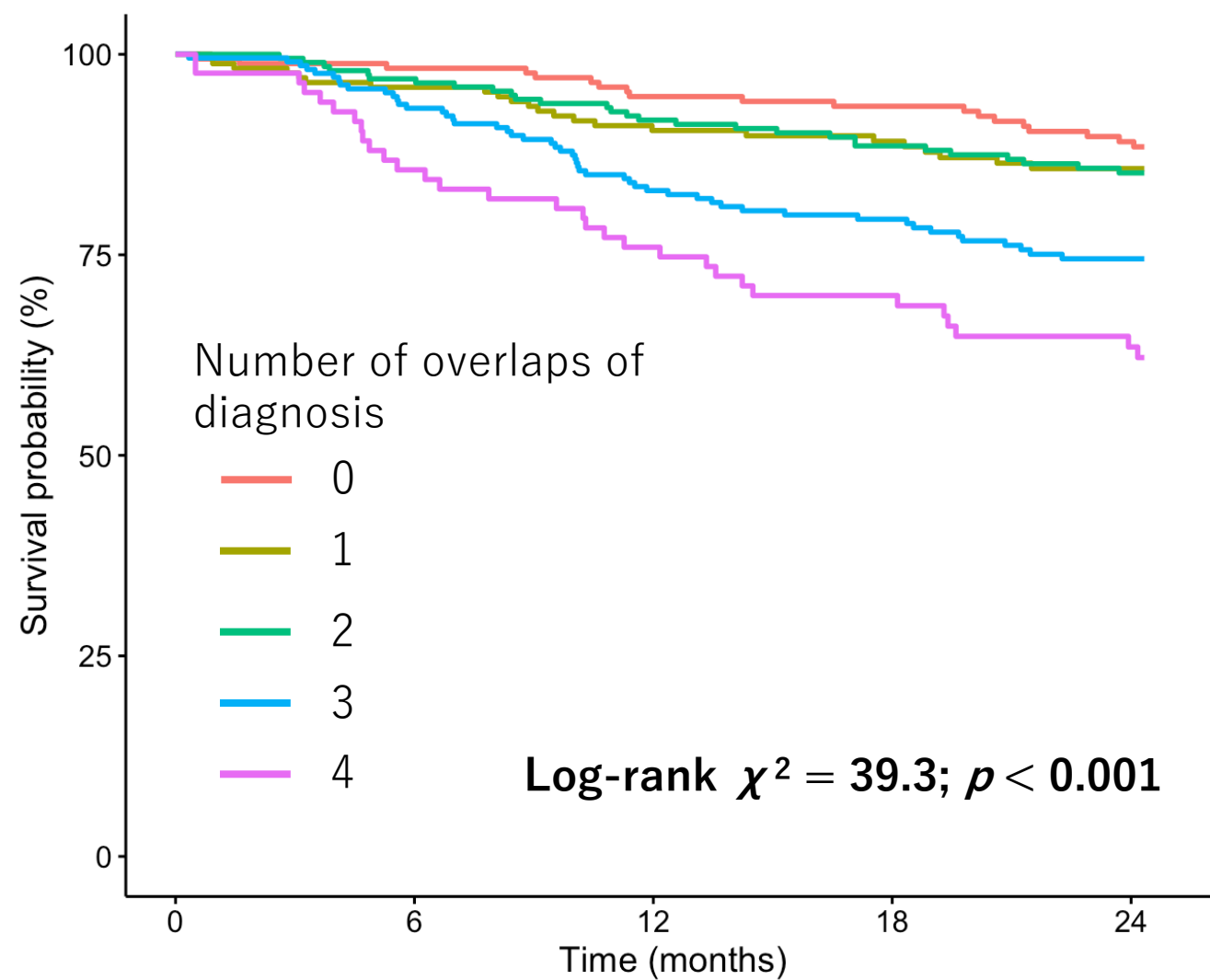

Number at risk

|   |     |     |     |     |     |
|---|-----|-----|-----|-----|-----|
|   | 0   | 6   | 12  | 18  | 24  |
| 0 | 173 | 167 | 161 | 153 | 139 |
| 1 | 172 | 163 | 148 | 131 | 120 |
| 2 | 198 | 190 | 176 | 162 | 146 |
| 3 | 216 | 193 | 168 | 148 | 129 |
| 4 | 87  | 71  | 63  | 55  | 48  |

Time (months)

Supplement: Supplementary file 2 — Figure S2. Kaplan–Meier survival curves of overall survival rate in patients divided into five groups based on the number of overlaps of cachexia evaluated by AWGC and Evans’ criteria, sarcopenia, and malnutrition.AWGC, Asia Working Group for Cachexia. [file JCSM-15-2660-s001.pdf]
