# Supplementary material for: Prevalence and Prognostic Value of Cachexia Diagnosed by New Definition for Asian People in Older Patients With Heart Failure
Source: J Cachexia Sarcopenia Muscle. 2024 Nov 5;15(6):2660–8. doi: 10.1002/jcsm.13610 (PMC11634483; doi:10.1002/jcsm.13610)
Supplement: Supplementary file 3 — Table S1. Patient characteristics. [file JCSM-15-2660-s002.docx]

**Supplemental Table S1: Patient characteristics**

|  | Missing　data, n (%) | Overall | Cachexia by the AWGC | |  | Cachexia by the Evans’s | |  | Sarcopenia | |  | Malnutrition | |  |
| --- | --- | --- | --- | --- | --- | --- | --- | --- | --- | --- | --- | --- | --- | --- |
|  |  |  | No | Yes |  | No | Yes |  | No | Yes |  | No | Yes |  |
|  |  | n = 861 | n = 223 | n = 638 | *p*-value | n = 549 | n = 312 | *p*-value | n = 684 | n = 177 | *p*-value | n = 386 | n = 475 | *p*-value |
| Heart rate [beats/min] | 0 | 69 [60–80] | 67 [60–77] | 70 [60–80] | 0.174 | 68 [60–79] | 70 [61–80] | 0.172 | 69 [60–80.0) | 70 [62–80] | 0.318 | 68 [60–78] | 69 [61–80] | 0.227 |
| Systolic blood pressure [mm Hg] | 0 | 112 [103– 125] | 113 [105–126] | 112 [102– 124] | 0.080 | 112 [104–126] | 112 [102– 122] | 0.067 | 112 [104–126] | 112 [100– 122] | 0.147 | 114 [105– 127] | 110 [100–124] | <0.001 |
| Diastolic blood pressure [mm Hg] | 0 | 62 [56–68] | 62 [58–70] | 61 [54–68] | 0.019 | 62 [56–69] | 60 [54–67] | 0.093 | 62 [56–68] | 60 [52–67] | 0.041 | 63 [58–70] | 60 [54–67] | <0.001 |
| NYHA class III/IV | 0 | 102 (11.8) | 20 (9.0) | 82 (12.9) | 0.122 | 63 (11.5) | 39 (12.5) | 0.655 | 80 (11.7) | 22 (12.4) | 0.788 | 38 (9.8) | 64 (13.5) | 0.101 |
| Comorbidities |  |  |  |  |  |  |  |  |  |  |  |  |  |  |
| Hypertension, n (%) | 0 | 614 (71.3) | 164 (73.5) | 450 (70.5) | 0.392 | 403 (73.4) | 211 (67.6) | 0.072 | 497 (72.7) | 117 (66.1) | 0.086 | 293 (75.9) | 321 (67.6) | 0.007 |
| Dyslipidemia, n (%) | 0 | 310 (36.0) | 88 (39.5) | 222 (34.8) | 0.211 | 206 (37.5) | 104 (33.3) | 0.218 | 250 (36.5) | 60 (33.9) | 0.512 | 161 (41.7) | 149 (31.4) | 0.002 |
| Diabetes mellitus, n (%) | 0 | 293 (34.0) | 86 (38.6) | 207 (32.4) | 0.097 | 194 (35.3) | 99 (31.7) | 0.283 | 239 (34.9) | 54 (30.5) | 0.267 | 135 (35.0) | 158 (33.3) | 0.598 |
| Coronary artery disease, n (%) | 0 | 295 (34.3) | 78 (35.0) | 217 (34.0) | 0.794 | 190 (34.6) | 105 (33.7) | 0.777 | 236 (34.5) | 59 (33.3) | 0.770 | 139 (36.0) | 156 (32.8) | 0.330 |
| Atrial fibrillation, n (%) | 0 | 384 (44.6) | 115 (51.6) | 269 (42.2) | 0.015 | 266 (48.5) | 118 (37.8) | 0.003 | 313 (45.8) | 71 (40.1) | 0.178 | 177 (45.9) | 207 (43.6) | 0.504 |
| Current smoker, n (%) | 3 (0.3) | 124 (14.5) | 26 (11.7) | 98 (15.4) | 0.168 | 77 (14.1) | 47 (15.1) | 0.678 | 90 (13.2) | 34 (19.3) | 0.039 | 55 (14.3) | 69 (14.6) | 0.923 |
| Medications |  |  |  |  |  |  |  |  |  |  |  |  |  |  |
| Beta-blocker, n (%) | 0 | 648 (75.3) | 179 (80.3) | 469 (73.5) | 0.044 | 419 (76.3) | 229 (73.4) | 0.339 | 511 (74.7) | 137 (77.4) | 0.459 | 291 (75.4) | 357 (75.2) | 0.938 |
| ACE inhibitor ARB, n (%) | 0 | 604 (70.2) | 164 (73.5) | 440 (69.0) | 0.199 | 397 (72.3) | 207 (66.3) | 0.066 | 484 (70.8) | 120 (67.8) | 0.442 | 280 (72.5) | 324 (68.2) | 0.167 |
| Loop diuretics, n (%) | 0 | 748 (86.9) | 186 (83.4) | 562 (88.1) | 0.075 | 472 (86.0) | 276 (88.5) | 0.299 | 594 (86.8) | 154 (87.0) | 0.954 | 328 (85.0) | 420 (88.4) | 0.136 |
| MRA, n (%) | 0 | 428 (49.7) | 115 (51.6) | 313 (49.1) | 0.519 | 270 (49.2) | 158 (50.6) | 0.680 | 338 (49.4) | 90 (50.8) | 0.734 | 186 (48.2) | 242 (50.9) | 0.420 |
| Mid upper arm circumference [cm] | 11 (1.3) | 24.0 [21.8–26.3] | 25.8 [23.8–27.8] | 23.3 [21.2–25.5] | <0.001 | 24.5 [23.0–27.0] | 22.6[20.2–24.8] | <0.001 | 24.4 [22.4–27.0] | 22.5 [20.6–24.0] | <0.001 | 25.7 [24.0–27.5] | 22.6 [20.5–24.2] | <0.001 |
| Triceps skinfold thickness [cm] | 23 (2.7) | 1.0 [0.7–1.6] | 1.2 [0.8–1.7] | 1.0 [0.6–1.4] | <0.001 | 1.0 [0.8–1.6] | 0.9 [0.6–1.5] | 0.002 | 1.0 [0.7–1.6] | 0.9 [0.6–1.2] | 0.002 | 1.2 [0.8–1.7] | 0.9 [0.6–1.2] | <0.001 |
| Mid upper arm muscle circumference [cm] | 23 (2.7) | 20.5 [18.3–22.3] | 21.8 [20.0–23.6] | 20.0 [17.9–21.8] | <0.001 | 21.0 [19.2–22.9] | 19.2 [16.8–21.2] | <0.001 | 20.8 [18.7–22.7] | 19.2 [17.3–20.8] | <0.001 | 21.8 [19.9–23.4] | 19.5 [17.6–21.0] | <0.001 |
| Appendicular skeletal muscle index [kg/m^2^] | 54 (6.3) | 7.0 [6.2–8.0] | 7.6 [6.7–8.6] | 6.8 [6.1–7.8] | <0.001 | 7.2 [6.4–8.2] | 6.6 [6.0–7.6] | <0.001 | 7.4 [6.6–8.4] | 6.0 [5.4–6.5] | <0.001 | 7.6 [6.8–8.5] | 6.6 [6.0–7.4] | <0.001 |
| Handgrip strength [kg] | 1 (0.1) | 20.5 [14.9– 26.6] | 26.0 [19.5– 31.7] | 19.0 [14.0– 24.1] | <0.001 | 22.0 [16.4– 29.4] | 17.2 [13.0– 22.2] | <0.001 | 21.0 [15.1– 28.4] | 17.7 [14.0– 21.9] | <0.001 | 22.3 [16.4– 29.2] | 18.6 [14.0– 24.0] | <0.001 |
| Short physical performance battery [point] | 12 (1.4) | 10 [7– 11] | 11 [8– 12] | 9.0 [7.0– 11.0] | <0.001 | 10.0 [7.0– 12.0] | 9.0 [6.0– 11.0] | <0.001 | 10.0 [7.0– 12.0] | 8.0 [6.0– 10.0] | <0.001 | 10.0 [7.0– 12.0] | 9.0 [7.0– 11.0] | 0.011 |
| Six-min walking test distance [m] | 43 (5.0) | 270 [180– 368] | 327 [208– 400] | 257 [170– 350] | <0.001 | 300 [195– 384] | 250 [162– 325] | <0.001 | 280 [195– 380] | 240 [150– 323] | <0.001 | 300 [191– 380] | 265 [180– 350] | 0.036 |
| median [interquartile range]; n, number (%)  NYHA, New York Heart Association; ACE, angiotensin converting enzyme; ARB, angiotensin receptor blocker; MRA, mineralocorticoid receptor antagonist | | | | | | | | | | | | | | |
