# Supplementary material for: Prevalence and Prognostic Value of Cachexia Diagnosed by New Definition for Asian People in Older Patients With Heart Failure
Source: J Cachexia Sarcopenia Muscle. 2024 Nov 5;15(6):2660–8. doi: 10.1002/jcsm.13610 (PMC11634483; doi:10.1002/jcsm.13610)
Supplement: Supplementary file 4 — Table S2. Cox regression analyses of the association of the coexistence of multiple diagnoses for the all‐cause death. [file JCSM-15-2660-s004.docx]

**Supplemental Table S2: Cox regression analyses of the association of the coexistence of multiple diagnoses for the all-cause death**

|  | Model 1 | | | Model 2 | | | Model 3 | | |
| --- | --- | --- | --- | --- | --- | --- | --- | --- | --- |
|  | Adjusted HR | 95% CI | *p*-value | Adjusted HR | 95% CI | *p*-value | Adjusted HR | 95% CI | *p*-value |
| Number of diagnoses |  |  |  |  |  |  |  |  |  |
| 0 | 1.000 | [Reference] |  | 1.000 | [Reference] |  | 1.000 | [Reference] |  |
| 1 | 1.255 | 0.680−2.316 | 0.465 | 1.258 | 0.681−2.326 | 0.461 | 1.184 | 0.640−2.190 | 0.589 |
| 2 | 1.226 | 0.680−2.209 | 0.496 | 1.201 | 0.665−2.169 | 0.542 | 1.039 | 0.573−1.885 | 0.898 |
| 3 | 2.172 | 1.270−3.715 | 0.005 | 2.037 | 1.188−3.495 | 0.010 | 1.643 | 0.949−2.842 | 0.076 |
| 4 | 3.612 | 2.025−6.442 | <0.001 | 3.433 | 1.913−6.163 | <0.001 | 2.681 | 1.480−4.857 | 0.001 |
| Model 1: age and sex  Model 2: model 1 + log-transformed B-type natriuretic peptide and estimate glomerular filtration rate  Model 3: model 2 + the Meta-analysis Global Group in Chronic Heart Failure score  HR, hazard ratio; CI, confidence interval. | | | | | | | | | |
